# Improving competence and safety in pain medicine: a practical clinical teaching strategy for students combining simulation and bedside teaching

**DOI:** 10.1186/s12909-021-02554-6

**Published:** 2021-02-25

**Authors:** Sandra Kurz, Jana Lohse, Holger Buggenhagen, Irene Schmidtmann, Rita Laufenberg-Feldmann, Kristin Engelhard

**Affiliations:** University Medical Center of the Johannes Gutenberg-University, Langenbeckstraße 1, 55131 Mainz, Germany

**Keywords:** Medical training, Multi-professional education, Simulated patients, Interactive medical training, Curriculum innovation, Bedside teaching

## Abstract

**Background:**

Pain is a devastating sensation and has to be treated immediately. Therefore, we developed a training program to improve the knowledge of medical students in the field of pain medicine. In the present study, the applicability and efficacy of this training program was tested.

**Methods:**

Half of the students attended first a training with simulated patients (SP) followed by bedside teaching (Group 1). Group 2 performed the training programs in reverse order. The evaluation based on standardized questionnaires completed by students (self-assessment) and all students took part in two practical examinations after the learning interventions.

**Results:**

This study included 35 students. The quality of the simulation was evaluated by the students with average grade 1.1 (1 = very good, 6 = very bad). The practical work on the ward with patients was rated with grade 1.4 of 6, the whole course with 1.1. Students of Group A were significantly better in the final examination (grade 1.7 vs. grade 2.2, *p* < 0.05). To rate the improvement of skills (self-assessment) we used a Likert Scale (1 = very certain, 5 = very uncertain). The following skills were similar in both groups and significantly better after the course: taking responsibility, expert knowledge, empathy, relationship building and communication.

**Conclusions:**

Training with simulated patients in combination with small-group teaching at the bedside with real patients achieves a dramatic increase in student competence. Students prefer learning from the simulation before bedside teaching and propose to include simulation into the curricular teaching of pain medicine.

**Supplementary Information:**

The online version contains supplementary material available at 10.1186/s12909-021-02554-6.

## Background

Experiencing pain is extremely stressful and unpleasant. Therefore, it is very important to train medical students to diagnose and treat patients with acute and chronic pain properly. To meet this challenge in Germany the cross-sectional field “Pain Medicine” was implemented in the curricular teaching of medical students. At the University Medical Center of Mainz the cross-sectional field “Pain Medicine Q14” has been included in the 8th semester of the medical school educational program since 2014, instructing about 200 students in pain medicine every semester [1–4]. However, due to the large number of medical students and limited university teaching-resources, teaching of detailed practical skills for students is still inadequate. A lack of communication skills results in inadequate, improperly treatment of patient with acute or chronic pain, therefore training in this regard is important [5–7]. It is meaningful and necessary to intensify the efforts to teach medical students especially in the practical aspects of pain medicine using specific practical training sessions [8–12].

We established a new elective course in order to fill the gap and thereby help students to acquire more competence and safety in treating patients with acute pain. The aim is to enable young physicians to start acute pain therapy autonomously in routine patients and to work in an interdisciplinary and professional team when the therapy becomes more difficult. Studies within the framework of medical training revealed a good effectiveness of simulation-based training on procedural and clinical skills, as well as aspects of non-technical skills [13, 14]. Therefore, we included simulated patients (SP’s) and patients on the ward (Clinic) in our course.

In spite of the well-known effectiveness and importance of simulation-based education prior to hands-on practice, reliable data on this topic is still sparse and simulation-based learning is still not implemented as a major teaching method in medical schools in Germany [15–18].^,^

The objective of the present study is to test, whether students, who have experienced training with simulated patients prior to contact with real patients, have better clinical skills than students who receive training in reverse order.

## Methods

**Fig. 1:**
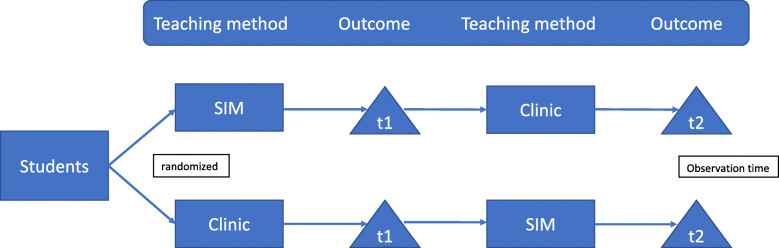
Study design with two groups

The study was designed as a prospective randomized study. Students with interests in pain medicine chose the elective course “Competence and Safety in Pain Medicine”. At the beginning of the course, the participating students were randomized in two groups. Group A received the simulation training first followed by bedside teaching, whereas Group B experienced bedside-teaching first followed by simulation training afterwards (Fig. 1). Both groups were assessed after both teaching units. We included 36 students in our study, of whom 35 could be evaluated. One student was unable to complete the course and was excluded from the study.

During the study, we measured the skills of students at different times through self-assessment and assessment by a teacher (supplementary file). We asked the students to fill out a first questionnaire with questions regarding age, gender, professional experience and course expectations. Further, they had to rate the improvement of skills in the self-assessment using a five-point rating scale where 1 equals very certain and 5 equals very uncertain to the statement, “To take on responsibility”, “Expert knowledge of pain medicine”, “Empathy”, “Relationship building with other people” and “Communication with patients”.

The training took place in small groups with three students trained with simulated patients, as well as learning in clinical situations with patients on a ward. The simulation patients were experienced amateur actors from our SP-team. Across the three courses they play the same role. Different simulated patients were used for each scenario and for the tests. Before the course, they completed a scenario- and a feedback- training. Each section ended with a peer-feedback by the observing students as well as a feedback by the simulated patient and the instructor. The course was scheduled for 4 days from 8:30 am to 03:00 pm. We tested at two different times to analyze knowledge acquisition in communication and clinical treatment. Day one and three were planned as training-days, day two and four as exam-days.

All students signed a consent form to allow a blinded collection of their data. A non-consent had no consequences for the students, meaning that participation in the simulation without data collection was possible. For the teaching of 12 students per week, we needed four instructors (two for the simulation and respectively one for acute pain medicine and chronic pain medicine) on day one and three. For the assessment on day two and four we needed two instructors.

The most important learning objectives of the course were, that students knew the criteria of a patient-oriented conversation and could exchange information interdisciplinary. Further, that students were able to demonstrate a physical examination of a pain patient. Students should also be able to arrange post-operative pain therapy according to the WHO level scheme. Another important objective was that students were familiar with various procedures for the treatment of postoperative pain (opioids s.c., Iv.-PCA, epidural anesthesia, peripheral nerve-catheters). Students should also know the clinical symptoms of an epidural haematoma and could diagnose it as a rare but important unwanted risk.

### Educational and assessment methods

Two training methods were applied in this study: training with simulated patients and clinical training on the ward. The learning objectives were defined by a team of specialists of pain medicine and experts for medical education (Master of Medical Education). Every student experienced both methods in alternate order.

#### Training with simulated patients (SIM)

The simulation session included two short lectures and three scenarios with simulated patients and feedback. Group A took part in this session at day one; group B at day three.

#### Teaching on the ward (clinic)

The clinical session on the ward included a practical part in acute pain medicine and a part in chronical pain medicine. A second time the six students were divided into groups of three students each. Half of the students had a session in acute pain medicine first, followed by a session in chronical pain medicine after a break and vice versa. Group B had this session on day one, group A on day three.

For both teaching methods, all instructors received a train-the-trainer instruction with reviewing the learning objectives and the methods by the course-responsible.

After the first intervention (group A simulation and group B clinic) the students completed the second questionnaire with self-assessment questions and they had a practical examination by treating a simulated patient with acute pain. The examiner evaluated the communication skills and therapy skills with a checklist and calculated a score for the performance.

On the third day, the students had the second intervention day (simulation or clinic) and the second examination with a scenario and third self-assessment questionnaire followed on day four.

The simulated patients of the examination days and the training days were different so that the students who were in the simulation group had no advantages by already knowing the actor.

Our study included interprofessional education. On the simulation day, physicians with experience in pain medicine instructed the students supplemented with a teaching lesson by a pain nurse. In both groups, the ratio of teaching with a physician and the pain nurse was comparable.

### Expected action and learning objections

Opening of the consultation and principles of communication:

- The student welcomes the patient and introduces her−/himself.
- The student consults patient’s condition.
- The student listens to the patient.
- The students let the patient finish speaking.
- The student pays attention to nonverbal signals expressing pain.

Therapy:

- The problem of the patient is detected.
- The diagnosis is provided and understandably communicated to the patient.
- The student takes a goal-leading decision (e.g. adequate therapy or change of the treatment).
- Response time is less than 30 s to start or change the therapy.
- The student focuses on pain therapy.
- The student assures the patient safety.
- The student describes the further course of treatment.
- The student waits until the patient is satisfied.
- The student chooses the right therapy.
- The student consideres other therapy options.
- The student documents the diagnosis and therapy.

Closure of the consultation:

- The student clarifies whether the patient has any questions.
- The student explains how to proceed and how to reach the contact person.

All of the items listed above were assessed using a checklist. The examinator rated in each case whether the item was performed well, indifferent or bad.

### Statistical analysis

Statistical analysis was performed using SPSS version 23. For the assessment of the learning success, we used the mentioned checklist. From all items we rated good (two points), indifferent (one point) or bad (zero points) and calculated a sum for communication skills and therapy skills and overall a school grade on a scale of one to six, one being very good.

To rate the improvement of skills in the self-assessment we used a five-point rating Scale (1 = very certain, 5 = very uncertain). The group differences were tested with the standard t-test for independent samples. The significance level was chosen as α= 0.05, no adjustment for multiple testing was applied.

### Ethical considerations

Students participated voluntarily. The tests and questionnaires treated anonymously and calculated according to randomized identification numbers. The local ethics committee of the Medical Association of Rhineland Palatine approved the study.

## Results

In total 35 students participated in the study. The students were medical students of the Johannes Gutenberg University of Mainz. Overall, 39% were male. From the participating students, 58% had no previous experience in medical occupations, 42% had previous experience (emergency rescue service or nursing). The median age of the students was 27 years (22 to 41 years).

### ***Results of the*** objective ***assessment***

The statistical analysis of the trial showed that students of Group A were significantly better in the final examination (1.7 vs. 2.2 out of 6 points, *p* < 0.05). From these results, we concluded that the simulation training (SIM) should precede the bedside training (Clinic) (Table 1).

**Table 1:** Observed grades by group in the second examination (grade on a scale of 1 to 6; 1 = very good)

	Group 1 (*n* = 18)	Group 2 (*n* = 17)	*P* value*
Mean ± Standard deviation	1,7 ± 0,8	2,1 ± 0,7	0,0043

* t-Test

**Fig. 2:**
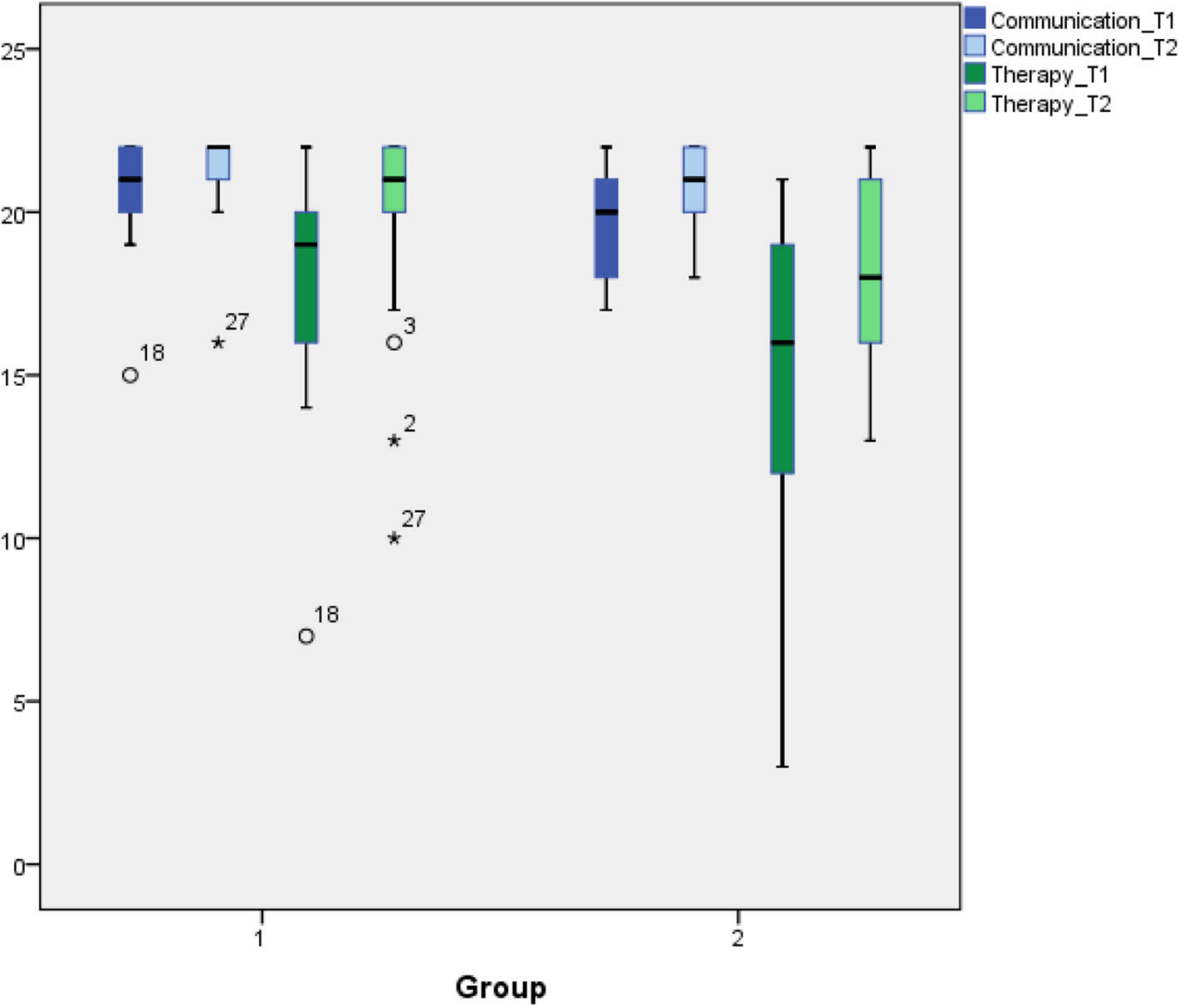
Skills in communication and choice of therapy

Regarding the values by clustering the items “communication” and “therapy (Fig. 2) we saw an increased score in examination two in both groups but without significant difference in the standard t-test.

### Results of the self-assessment

Table 2 shows the self-assessment of personal clinical competence after the complete course compared to the situation before the students received the training. After the training, all students felt more competent regarding all topics.

**Table 2:** The acquisition of competence in the self-assessment before and after the course based on a Likert Scale of 1–5 (1 = very competent, 5 = not competent). Averages of values on Likert scale (± Standard deviation)

	before the course	after the course	*p*-Value
To overtake responsibility	2.8	1.8	*p* < 0.05
Expert knowledge of pain medicine	3.2	2.0	*p* < 0.05
Empathy	1.8	1.4	*p* < 0.05
Relationship building with other people	1.9	1.6	*p* < 0.05
Communication with patients	2.1	1.5	*p* < 0.05

### Results of the evaluation

The simulation was evaluated by the students with a mean of 1.1 (1 = very good, 6 = very bad). The students enjoyed the practical work on the ward with patients with acute and chronical pain (both rated with 1.4 of 6 points). The examination was rated positive with 1.3 (out of 6 points). Overall, students were highly impressed by the course concept and marked the course with a 1.1.

## Discussion

The importance of an adequate training in the treatment of patients suffering of acute or chronic pain comes more and more to the fore of national and international medical schools and hospitals. The complex treatment of physical and psychosocial issues requires more than just medical knowledge.

Medical education based on simulation has been implemented in the 1950, but simulation-based soft-skill training in particular is still in the early phase of its development [15].

Simulated-based education is crucial for optimal patient treatment and became popular in the last few years [17, 19]. In order to improve the treatment of patients suffering from pain, we established an intensive training course for students, who were interested in this topic and who wanted to increase their competence in interdisciplinary and multi-professional work. This course supplemented the curricular cross-disciplinary subject with high ratio of practical training for interested students. We decided to train the students using simulation and clinical bedside teaching. As it was unclear, whether we should first perform the bedside training or the simulation, the present study was designed to compare both teaching methods and to evaluate the optimal teaching method for clinical skills. Until now, only a few studies have evaluated the transfer of knowledge and skills from the simulation to the patient [20].

As successful and empathic communication has a positive influence on patient’s satisfaction and safety we should focus on these aspects in teaching [21]. For teaching communication and other soft-skills the method of using SPs seems to be useful. Especially getting feedback from the group, the SP and the teacher in the debriefing has a high impact for the learning success. To find the right way to communicate with patients with pain and to treat them is challenging, so the feedback can help to reflect how to handle these patients during real clinical situations [22–27].

As expected, students of both groups showed a very good learning success. The grades in the second examination suggest that the sequence to first train with simulated patients, followed by clinical training could be superior. Therefore, the authors conclude that it is beneficial to teach the simulation first before the practical training, which is also preferred by the students. However, if organizational reasons require a change in order, this is possible because there still exist a learning effect.

We and other authors are convinced that a simulation before realistic training have many advantages [28]. There are ranges of simulation methods including task trainers, mannequin-based simulation, virtual reality, screen-based simulations as well as simulated patients that can be used before handle real patient [29]. On the other hand, it has to be considered that students tend to overrate their clinical abilities and knowledge improvement after simulation training [30]. Nikendei et al. evaluated a training for ward round skills, integrating simulated patients and revealed, that training with simulated patients is well accepted and is very useful in order to prepare students for contact with patients during their final year at medical school [31, 32].

Clerkships are widely considered as favorable learning environment, in which students are able to learn in realistic situations, but often an adequate supervision and feedback is lacking. For the success of practical training it is important to have small groups on the ward and that the students take over an active and not only an observing role [33]. In our study, we had the same care key of 3 to 1 at the ward as in the simulation, so the students were adequately supervised during all situations. Our results show that the students liked and benefited from both teaching-methods.

The motivation and knowledge of the trainer is also very important for successful teaching. Our trainers were all experienced in pain medicine and were dedicated to education. They all completed a train-the-trainer course before the study to adjust the learning goals and methods. The interprofessional team of instructors in the present study, physicians and pain nurses, most likely further improved the quality of the training, communication, and teamwork skills [34, 35]. We had to limit the capacity for this course format to a maximum of 12 students per week. To integrate practical training for a whole cohort we have to think about alternative or supplementary methods to the simulation, possibly using virtual patients or blended learning. The use of e-learning modules is an engaging way to teach medical skills and competencies or to enable students to develop appropriate attitudes towards their future professional role [36–38]. There are a few platforms developing scenarios with virtual patients to learn clinical decision-making skills. Studies showed that e-learning with virtual patients and simulated patients are a valuable addition to clinical teaching [39–44].

Performance in the scenario-training course and the teaching on the ward was given to the students as part of a debriefing. Optimally, the performance review should be supplemented by an OSCE (Objective Structured Clinical Examination) with about eight stations to verify the acquired practical skills with the correct method. For 4 days of teaching, we employed two or four instructors per day and the personnel expenses were already very high. For smaller groups and close contact, the method of continuous assessments with individual practical scenarios is also reasonable instead of an OSCE. Not only the implementation but also the development of OSCE stations is very time-consuming and a platform for sharing OSCE-station can be helpful [45].

### Limitations of the study

There are a few limitations to this educational innovation study. The small sample sizes may hamper to reach conclusions on the effectiveness of the two different learning types. To include a pretest would be the superior method, but the additional expenditure would be too high.

## Conclusion

A training with simulated patients followed by a bedside teaching is a very effective educational model for intensified teaching in order to improve the skills of students to treat patients with pain. The student’s ability to communicate and treat pain patients as well as their satisfaction was high when they were instructed first in a simulated environment followed by real bedside teaching. The downside of this intensified training are the high costs due to the requirement of multiple instructors. Nevertheless, the improved competence of the future doctors to treat patients who are suffering from pain might justify this investment.

## Supplementary Information

**Additional file 1.** Questionnaire. The questionnaire used in your study was developed for this study and is included in the additional files.

**Additional file 2.**

## Data Availability

The datasets used and analysed during the current study are available from the corresponding author on reasonable request.

## Abbreviations

SIM: Simulation with simulated patients
Clinic: Bedside Teaching
t1 and t2: Examination (test) at two different times
