# Supplementary material for: Improving competence and safety in pain medicine: a practical clinical teaching strategy for students combining simulation and bedside teaching
Source: BMC Med Educ. 2021 Feb 25;21:133. doi: 10.1186/s12909-021-02554-6 (PMC7905916; doi:10.1186/s12909-021-02554-6)
Supplement: Supplementary file 1 — Additional file 1. Questionnaire. The questionnaire used in your study was developed for this study and is included in the additional files. [file 12909_2021_2554_MOESM1_ESM.docx]

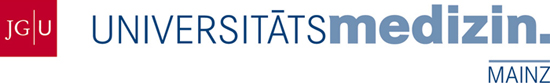


**Barcode**

**Final questionnaire of the study**

**Competence and safety in pain medicine**

Dear students!

As part of the KUSS project, we want to improve teaching in the field of pain therapy. We need your help to do this! The results of this questionnaire will make a significant contribution to the design of this curriculum, and to the future design of medical education in Mainz.

**Participation is voluntary and all data collected will be treated confidentially**

1. Have your expectations regarding the course been met?

Yes  no

Why / Why not?

1. How do you assess your competence after completing the course in the following competences?

**Very competent**  **Very incompetent**

**Taking responsibility**

**Expertise**

**Empathy**

**Building relationships with other people**

**Communication skills**

**... with the patient**

**… with colleagues**

1. What was the role model function of the tutors supervising you in the elective subject KUSS with regard to …

**Very good** **very bad**

**Taking responsibility**

**Expertise**

**Empathy**

**Building relationships with other people**

**Communication skills**

... **with the patient**

… **with colleagues**

|  | **Applies to** |  | **Neither nor** |  | **Doesn't**  **apply** |
| --- | --- | --- | --- | --- | --- |
|  | **+ +** | **+** | **O** | **-** | **- -** |
| **Structure and accompanying materials** | | | | | |
| 1. The concept and structure of the event were clear. |  |  |  |  |  |
| 1. The event brought me many new content insights. |  |  |  |  |  |
| 1. The learning objectives described at the beginning of the event have been met. |  |  |  |  |  |
| 1. The event-accompanying materials facilitate the understanding of the content/substance. |  |  |  |  |  |
| **Design / Didactics** | | | | | |
| 1. It was important to the lecturers that the students learn something. |  |  |  |  |  |
| 1. The learning content was sufficiently illustrated with comprehensible examples. |  |  |  |  |  |
| 1. The lecturers always followed a clear, comprehensible, common thread |  |  |  |  |  |
| 1. The lecturers are links to previous knowledge from previous events. |  |  |  |  |  |
| 1. The lecturers spoke clearly and clearly |  |  |  |  |  |
| 1. The exercise tasks contributed to the understanding of the event |  |  |  |  |  |

|  | **Applies to** |  | **Neither nor** |  | **Doesn't**  **apply** |
| --- | --- | --- | --- | --- | --- |
|  | **+ +** | **+** | **O** | **-** | **- -** |
| **Interaction** | | | | | |
| 1. The participants were encouraged to critically engage with the contents of the event. |  |  |  |  |  |
| 1. The lecturers created an atmosphere in which the participants could express questions and comments about the material without hesitation. |  |  |  |  |  |
| 1. The lecturers answered the participants' questions in a comprehensible way |  |  |  |  |  |

| **Assessment of individual teaching methods by school grade** | | | | | | |
| --- | --- | --- | --- | --- | --- | --- |
|  | **School grades** | | | | | |
|  | **1** | **2** | **3** | **4** | **5** | **6** |
| Lecture Acute pain |  |  |  |  |  |  |
| Lecture Chronic Pain |  |  |  |  |  |  |
| Simulation with actors |  |  |  |  |  |  |
| Workshop |  |  |  |  |  |  |
| Clinical use in acute pain |  |  |  |  |  |  |
| Clinical use in chronic pain therapy |  |  |  |  |  |  |
| Learning success checks with actors |  |  |  |  |  |  |
| Workshop Analgesia in children |  |  |  |  |  |  |
| Workshop BTM Recipe |  |  |  |  |  |  |

**Free text evaluation:**

I liked:

I didn't like it:

I give the course the school grade as a whole:
