# Supplementary material for: Improving competence and safety in pain medicine: a practical clinical teaching strategy for students combining simulation and bedside teaching
Source: BMC Med Educ. 2021 Feb 25;21:133. doi: 10.1186/s12909-021-02554-6 (PMC7905916; doi:10.1186/s12909-021-02554-6)
Supplement: Supplementary file 2 — Additional file 2. [file 12909_2021_2554_MOESM2_ESM.docx]

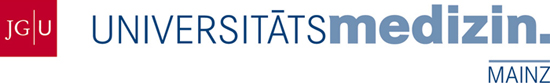


**Barcode**

**Survey questionnaire**

**Competence and safety in pain medicine**

Dear students!

As part of the KUSS project, we want to improve teaching in the field of pain therapy. We need your help to do this! The results of this questionnaire will make a significant contribution to the design of this curriculum, and to the future design of medical education in Mainz.

**Participation is purely voluntary and all data collected will be treated confidentially**

1. What motivated you to register for this course?
2. What are your expectations for the course?

continue at PART 2

1. How do you currently assess your expertise in pain medicine?

**Very competent**  **Very incompetent**

**Taking responsibility**

**Expertise**

**Empathy**

**Building relationships with other people**

**Communication skills**

**... with the patient**

**… with colleagues**

1. **The development of these competences has been supported in your medical studies**:

**Very supported not supported**

**Taking responsibility**

**Expertise**

**Empathy**

**Building relationships with other people**

**Communication skills**

**... with the patient**

**... with colleagues**

**PERSONAL DATA**

1. How old are you?

Jahre

1. Your gender is

Männlich Weiblich

1. You want to become an internist, surgeon, pediatrician, anesthesiologist, etc.)

 Weiß ich nicht

1. Do you have vocational training?

Ja Nein

1. If so, which ones?

1. You have medically relevant previous experience in the form of (multiple nominations possible):

Rescue service  Nursing

ERC – Course (Reanimation training)

Others:
